# Effect of immersion and thermocycling in different beverages on the surface roughness of single- and multi-shade resin composites

**DOI:** 10.1186/s12903-023-03069-w

**Published:** 2023-06-07

**Authors:** Aiah A. El-Rashidy, Omar Shaalan, Rasha M. Abdelraouf, Nour A. Habib

**Affiliations:** 1 Biomaterials Department, Faculty of Dentistry, Cairo University, Cairo, 11562 Egypt; 2 Conservative Dentistry Department, Faculty of Dentistry, Cairo University, Cairo, 11562 Egypt

**Keywords:** Single-shade, Multi-shade, Surface roughness, Stylus profilometer, AFM

## Abstract

**Background:**

Resin composite restorations are highly esthetic restorations, which should have and maintain high surface polish. However, esthetic restorations are subjected to different beverages at variable temperatures, which may affect their surface roughness. This study aimed to evaluate the surface roughness of single-shade (Omnichroma) and multi-shade (Filtek Z350XT) composite materials, following aging by immersion and thermocycling in different beverages, simulating one year of clinical service.

**Methods:**

Thirty specimens of each material were prepared and divided into 6 subgroups (n = 5). In each material, the grouping of the specimens was as follows: the first subgroup was the as-prepared specimens stored dry without immersion or thermocycling. The second, third, and fourth subgroups were immersed in saliva, tea, and red wine, respectively, for 12 days at 37 °C. The fifth and sixth subgroups were thermocycled for 10,000 cycles, in tea (the fifth between 37 and 57 °C) and in red wine (the sixth between 37 °C and12°C). The resultant surface roughness was measured by two different methods, stylus profilometer and atomic force microscopy (AFM). Intergroup comparison was performed using independent t test, while intragroup comparison was performed using one-way analysis of variance (ANOVA) followed by Tukey’s post-hoc test.

**Results:**

Intergroup comparison between both composites showed no statistically significant differences in all groups using the stylus profilometer roughness measurements (P>0.05), while the AFM measurements showed significant difference (P ≤ 0.05) within all storage media except the as-prepared control (P = 0.0645), where nanofilled Filtek Z350 XT showed lower nano-roughness. Intragroup comparison data were variable, depending on the material, aging conditions, and roughness assessment tool. However, the resultant average surface roughness (R_a_) values in all groups did not exceed the threshold value of R_a_ 0.2 μm.

**Conclusions:**

Both resin composites attained and retained a clinically acceptable surface finish after immersion and thermocycling in different beverages.

## Introduction

Esthetics in dentistry has become of prime importance nowadays, where restorations of high esthetic qualities have become an important requirement by patients, especially the restoration of maxillary anterior teeth [1]. One of the main points that contributes to esthetic success of resin composite restorations is their surface quality, which depends on the gloss and smoothness of the restoration [2–4].

Ideally, resin composite restorations should have and maintain high color stability, surface smoothness [5], dimensional stability [6], marginal integrity [7], wear resistance [8], etc. Maintaining surface smoothness of resin composite restorations is an important requirement, as rough surfaces contribute to increased staining susceptibility and external discoloration of the restoration, plaque accumulation, gingival irritation, periodontal disease, and recurrent caries [9, 10]. Several in vivo studies suggested that the threshold surface roughness for bacterial plaque retention was 0.2 μm [11], while a clinical study reported that a change of mean surface roughness at about 0.3 μm could be detected by most of the patients by tip of the tongue [12].

Surface roughness of composite resins is mainly affected by (a) the size, hardness, and amount of fillers, (b) resin matrix composition, and (c) bonding between fillers and resin matrix [2, 13, 14]. Increasing the filler content while reducing the filler size resulted in reduction in the wear rates and improved surface smoothness of resin composite restorations [13, 15].

Restorations placed in the oral environment are constantly subjected to thermal fluctuations due to the intake of foods and beverages at different temperatures [16]; however, few reports are available on how the temperature of these foods and beverages could affect composite resins, particularly regarding their surface roughness [16]. Thermocycling may increase water sorption, which could erode the surface of resin composites through the plasticizing effect of water and the hydrolysis of silane coupling agents [17, 18]. In addition, the pH of beverages (such as tea, coffee, wine, and soda drinks) could greatly affect the surface integrity of the restoration. Acidic pH solutions could cause hydrolysis and degradation of the polymer matrix [19, 20]. Moreover, alcohol in wine may act as a plasticizer of the polymer matrix, causing softening and degradation of the polymer [21], resulting in increased erosion and surface roughness in resin composites. Red wines were shown to result in increased surface roughness and erosion of nanohybrid and nanofilled resin composites, as compared to white wines, due to their higher alcohol concentration [22]. In addition a recent systematic review by Paolone et al. [23] reported that cigarette smoke, whether conventional or electronic cigarettes, significantly affect the color stability of resin composites. Cigarette smoke was also reported to affect the surface roughness of various dental resin composites materials [24–27]. Previous studies reported significant increase in the surface roughness of single-shade composite (Omnichroma) following exposure to simulated gastric acid challenge [20], while short term storage in coffee or tea solutions for 48 h or bleaching with 30% hydrogen peroxide did not affect its surface roughness [28, 29]. Based on the previous mentioned studies, the effect of longer staining durations, different beverages, and artificial aging protocols on the surface roughness of single-shade composite (Omnichroma) is still rather limited.

The oldest and most used surface parameter is the average roughness (R_a_), also known as the center line average (CLA) [30]. The R_a_ value is defined as the arithmetic average height, or the average absolute deviation of the roughness irregularities from the mean line over one sampling length [31].

Surface roughness measurement can be made using either quantitative or qualitative methods. Among the quantitative methods, surface profile analysis (e.g., stylus profilometer) and atomic force microscopy (AFM) are the most common measuring methods [30]. Hence, comparing and supporting profilometry findings with AFM measurements can lead to more accurate results [32].

There is insufficient data in the literature on the effect of thermocycling of drinks (tea and red wine) in their actual daily use temperatures and immersion on the surface roughness of single-shade resin composites as compared to the widely investigated multi-shade resin composites. Therefore, it was found beneficial in the current study to investigate the combined effect of different artificial aging protocols (immersion and thermocycling) in different solutions (artificial saliva, tea, and red wine), simulating one year of clinical service, on the surface roughness of two resin composite types (single-shade and multi-shade), and comparing the resultant surface roughness to the critical value reported in the literature (0.2 μm). The null hypotheses tested are that there will be no difference between surface roughness of both composites and that there will be no difference between different storage methods for both composites.

## Materials and methods

Two different composite materials were used in the present study, single-shade composite (Omnichroma Tokuyama Dental, Tokyo, Japan) and multi-shade composite (Filtek Z350 XT 3 M ESPE, Minnesota, USA). Materials used in the current study are described in Table 1.

**Table 1:** Materials’ manufacturer, composition, shade and lot number

Product	Manufacturer	Filler Type	Filler Content		Matrix Composition	Shade	Lot
			wt%	vol%			
Omnichroma	Tokuyama Dental, Tokyo, Japan	Uniform size supra nano-spherical fillers (260 nm spherical silica and zirconia)	79%	68%	TEGDMA* UDMA**,	Universal	016E21
Filtek Z350 XT	3 M ESPE, Minnesota, USA	Non-agglomerated/non-aggregated 20 nm silica filler and 4 to 11 nm zirconia filler, and aggregated zirconia/silica cluster filler (comprised of 20 nm silica and 4 to 11 nm zirconia particles)	78.5%	63.3%	TEGDMA*, UDMA**, Bis-GMA***, PEGDMA†, Bis-EMA††	A2B	NC93014

*TEGDMA = triethylene glycol dimethacrylate, **UDMA = urethane dimethacrylate,*** BisGMA = bisphenol A diglycidildimethacrylate, †PEGDMA polyethylene glycol dimethacrylate, ††Bis-EMA = Ethoxylatedbisphenol A dimethacrylate

### Sample size calculation

In a previous study by Nithya et al. in 2020 [33], the surface roughness within nanofilled resin composite (Filtek Z350XT) was normally distributed with standard deviation 0.104. By adopting a small Cohen’s d effect size of 0.2 as a difference between single-shade resin composite (Omnichroma) and multi-shade resin composite (Filtek Z350XT), we needed to study 5 experimental specimens per each subgroup to be able to reject the null hypothesis that the surface roughness means of the experimental and control groups were equal. The Type I error probability associated with this test of the null hypothesis was 0.05 and type 2 error probability was 0.2 with a power of 80%. Sample size was calculated using PS Power and Sample, version 3 for Windows (William D. Dupont and Walton D. Plummer) using independent t test.

### Specimens’ preparation

A total of 60 specimens were prepared, 30 specimens for each material, using a 1-mm thick and 8-mm diameter Teflon mold. After the composite resins were placed in the molds, Mylar strips and microscope glass slides were applied over the top surface of the composite resins using finger pressure. Each sample was light-cured through the Mylar strip and the glass slide for 20 s using a light-emitting diode (LED) curing unit (Mini LED, Satelec, Acteon, France), at a light intensity of 1,000 mW/cm^2^ from the top and bottom surfaces. The light intensity of the LED curing unit was checked with a spectroradiometer (Demetron Research Corp. USA). The flat smooth surface of the tested specimens was produced through polymerization of the pressed resin composite against a Mylar strip under pressure of a glass slide.

### Specimens aging

After preparation, specimens were divided into two groups, with 30 specimens for each material, then each group was subdivided into 6 subgroups (n = 5) according to the aging protocol and storage media. Grouping of the specimens was as follows: the first group was the as-prepared specimens, stored dry without immersion or thermocycling. The second, third and fourth groups of both materials were stored at constant temperature of 37 °C for 12 days, where the second group was stored in artificial saliva, the third group was stored in tea (Lipton Yellow Label; Unilever; UK) and the fourth group was stored in red wine (Omar El Khayam; Egypt). Every 3 days, specimens were rinsed with distilled water and immersed in fresh solutions to avoid any bacterial or fungal contamination [34, 35]. The fifth and sixth groups were thermocycled for 10,000 cycles over a dwell time of 60 s and a transfer time of 10 s at temperature range of the common average drinking temperature of tea (between 57 °C and 37 °C) and red wine (between 12 and 37 °C). The study was conducted to simulate one-year of clinical service, where immersion for 12 days in staining solution is equivalent to one year of beverages consumption [36], while in vitro thermocycling for 10,000 cycles simulates one year clinically [36, 37].

### Stylus profilometer roughness evaluation

The average surface roughness (R_a_, in µm), of the specimens was measured using contact mode surface profilometer (TR 220 Surface Roughness Tester, TIME Group, Pittsburgh, PA, USA), using a cut-off value of 0.8 mm and a range of 40 μm. Three measurements were recorded for each specimen and an average surface roughness (R_a_) was determined for each specimen.

### AFM roughness evaluation

Three-dimensional (3D) roughness profiles were assessed with contact mode AFM (5600LS Agilent Technology Company). Representative samples from each group were examined, three areas were randomly selected from each sample for R_a_ measurement, and AFM images were taken. Condition of measurement was as follows: size 200 × 200 nm, speed 0.71 inch/sec, I Gain = 2 and P Gain = 4 using contact mode.

### Statistical analysis

Data was analyzed using Medcalc software, version 19 for Windows (MedCalc Software Ltd, Ostend, Belgium). Data showed normal distribution using Kolmogrov Smirnov test and Shapiro Wilk test. Continuous data were described using mean and standard deviation. Intergroup comparison between continuous data was performed using independent t test, while intragroup comparison was performed using one-way analysis of variance (ANOVA) followed by Tukey’s post-hoc test. Partial correlation was used to correlate between stylus profilometer and AFM methods to assess surface roughness under the influence of material type and storage method. A P value less than or equal to 0.05 was considered statistically significant and all tests were two tailed.

## Results

### Stylus profilometer roughness measurement

The surface roughness measurements obtained using the stylus profilometer are shown in Table 2. Intergroup comparison between both composites have shown no significant differences in all groups (P>0.05) within each storage medium. Using one-way ANOVA, the intragroup comparison of storage media within the single-shade group showed a significant difference in the surface roughness resulting from storage in different immersion media (P < 0.001*). The artificial saliva produced the highest surface roughness, while storage in tea and red wine (immersion and thermocycling) induced the least roughness value, and the as-prepared control group showed intermediate roughness values. However, within the multi-shade group, there was no statistically significant difference between different storage media (P = 0.788).

**Table 2:** Mean and standard deviation of surface roughness of both materials within each storage medium measured with stylus profilometer

Material Storage	Single-shade composite		Multi-shade composite		P value
	Mean (µm)	SD	Mean (µm)	SD	
Control	0.14^b^	0.04	0.13	0.05	P = 0.6145
Storage Saliva	0.20^a^	0.02	0.18	0.13	P = 0.6275
Storage Tea	0.07^c^	0.02	0.11	0.08	P = 0.3500
Storage Wine	0.08^c^	0.01	0.15	0.10	P = 0.1500
Tea thermocycling	0.10^c^	0.02	0.17	0.16	P = 0.3590
Wine thermocycling	0.08^c^	0.01	0.10	0.08	P = 0.6642
P value	P < 0.001*		P = 0.788		

Means with different letters in the same column indicate statistically significance difference, while means in the same row have no letters as they are two groups only. *Corresponds to statistically significant difference

### AFM roughness evaluation

**Fig. 1:**
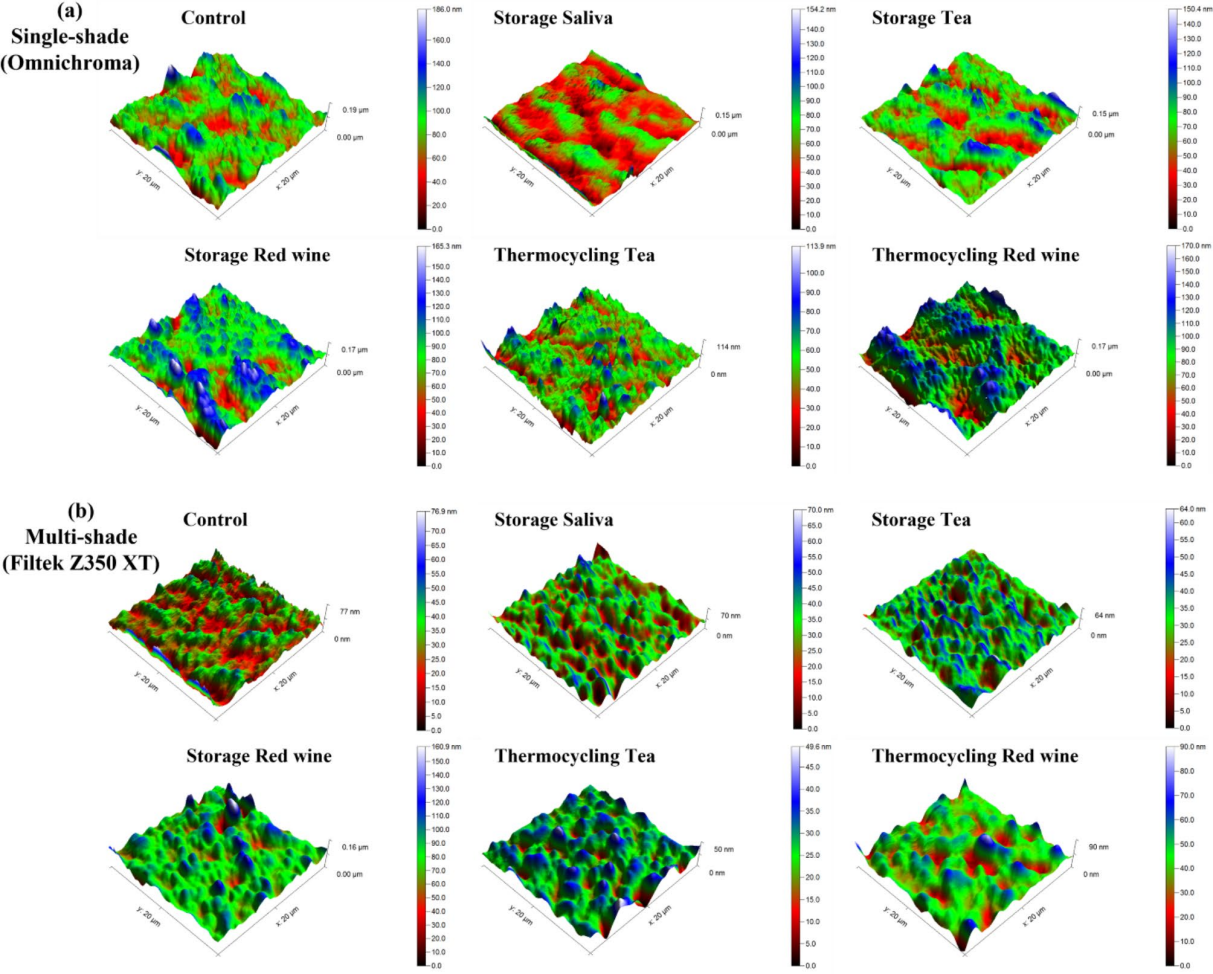
The 3D images of surface topography by AFM; (a) Single-shade (Omnichroma), (b) Multi-shade (Filtek Z350 XT)

The surface roughness measurement results obtained using the AFM are shown in Table 3. Intergroup comparison between both composites showed no significant difference within the control group (P = 0.0645), while it showed significant difference within the other groups (P ≤ 0.05) within each storage medium. Using one-way ANOVA, intragroup comparison of storage media within the single-shade group showed no statistically significant difference (P = 0.528), while within the multi-shade group there was statistically significant difference between different storage media (P = 0.005). Figure 1 shows the 3D images of surface topography obtained for different groups of single-shade and multi-shade composites.

**Table 3:** Mean and standard deviation of surface roughness of both materials within each storage medium measured with AFM

Intervention Storage medium	Single-shade composite		Multi-shade composite		P value
	Mean (nm)	SD	Mean (nm)	SD	
Control	2.05	0.20	1.35^a^	0.44	P = 0.0645
Saliva	2.00	0.09	0.53^b^	0.10	P < 0.0001*
Incubation Tea	1.82	0.16	0.49^b^	0.13	P = 0.0004*
Incubation red wine	2.25	0.74	0.96^ab^	0.25	P = 0.0459*
Thermocycling tea	2.29	0.34	0.70^ab^	0.23	P = 0.0025*
Thermocycling red wine	1.85	0.22	1.27^a^	0.28	P = 0.0480*
P value	P = 0.528		P = 0.005*		

Means with different letters in the same column indicate statistically significance difference while means in the same row have no letters as they are two groups only. *Corresponds to statistically significant difference

### Correlation between stylus profilometer and AFM data

There was negligible correlation between both methods in assessment of surface roughness (Correlation coefficient r = 0.2246, P = 0.2016).

## Discussion

Resin composite is a commonly used esthetic restoration, yet it is subjected to several oral fluids and beverages which may affect its surface roughness [22, 38, 39]. In the current study, the surface roughness of two resin composites (single-shade and multi-shade) was evaluated following aging in artificial saliva, tea and red wine using stylus profilometer and AFM. The stylus profilometer results showed no significant difference in the surface roughness between the two materials in the different storage media (P>0.05); therefore, the first null hypothesis was accepted. On the other hand, the AFM revealed significant difference between the two materials after artificial aging (P ≤ 0.05); therefore, the first null hypothesis was rejected. There was significant difference between storage media within the single-shade group using stylus profilometer and within the multi-shade group using AFM (P ≤ 0.05); therefore, the second null hypothesis was rejected. On the other hand, there was no significant difference between storage media within the multi-shade group using stylus profilometer and within the single shade group using AFM (P>0.05); therefore, the second null hypothesis was accepted.

The difference between stylus profilometer and AFM may be attributed to the low sensitivity of the stylus profilometer as a measuring tool compared to the AFM. The main limitation of stylus profilometer is the actual tip radius, which acts as mechanical filter unable to accurately detect features smaller than the tip radius [40]. Therefore, the stylus profilometer could not detect the nano-roughness. However, the AFM allowed the visualization of the surface topography of resin composite at a high spatial resolution, offering more detailed surface information than the profilometer [32]. There was negligible correlation between the two measuring methods, and this may explain the difference in the surface roughness values recorded by the stylus profilometer compared to that measured by the AFM for the same specimens. The mechanical stylus profilometer had an average resolution of 5 μm in x-y axis and 0.01 μm in z axis, while the AFM had higher resolution of ≈ 0.2 μm and 1.5 pm in x-y and z axes, respectively [41]. The stylus profilometer measured a larger area and provided information about micro-roughness, but the AFM scanned a smaller area with higher resolution, providing data at the nano-scale level which could not be detected by the stylus profilometer [32]. Therefore, they could be used simultaneously to give a comprehensive vision of the surface roughness.

The AFM showed no significant difference between the as-prepared specimens of the two materials without artificial aging. This may be attributed to the standardization of the specimens̕ preparation procedure [42]. Mylar strips are reported to produce optimally smooth surfaces as compared to different finishing and polishing systems [43–45]. This eliminated any variables during finishing and polishing that might interfere with the obtained smooth surfaces of the tested materials and avoided the introduction of any scratches and cracks which would increase the surface roughness [44], [46]; therefore, the two materials were evaluated in a more standardized manner. After aging in the different storage media, the AFM revealed that the multi-shade resin composite (Filtek Z350 XT) showed lower nano-roughness compared to the single-shade resin composite (Omnichroma). This may be due to the difference in the filler size present in the two materials. The multi-shade had relatively smaller sized fillers (20 nm silica filler and 4 to 11 nm zirconia filler) compared to the larger filler size of the single-shade (260 nm spherical silica and zirconia). In addition, the organic matrix of the multi-shade had Bis-GMA in its composition, which has lower susceptibility to acidic erosion and shows lower solubility compared to UDMA alone [47], [48].

It was previously reported that surface roughness above 0.2 μm resulted in increased bacterial accumulation and plaque adhesion. It was recommended to reduce the surface roughness below this value. The R_a_ = 0.2 μm was termed as "critical or threshold value̎ [18], [49]. In the present research, the roughness values detected by both the stylus profilometer and the AFM did not exceed the threshold value (0.2 μm) and were considered clinically accepted. This may be attributed to the filler size of the two examined materials. The single-shade resin composite contained uniform size spherical silica and zirconia fillers, 260 nm in size. These fillers, being larger than 100 nm, are considered submicron-sized particles and are described by the manufacturers as supra nano-fillers. However, the nanofilled multi-shade composite contains non-agglomerated/non-aggregated 20 nm silica filler and 4 to 11 nm zirconia filler, and aggregated zirconia/silica cluster filler (composed of 20 nm silica and 4 to 11 nm zirconia particles).

Regarding the intragroup comparison of storage media within single-shade, the stylus profilometer showed that there were significant differences in the micro-surface roughness resulting from storage in the different immersion media. The artificial saliva led to the highest surface roughness. This may be due to the degrading effect of saliva on the UDMA and TEGDMA, which were the only two components of the organic matrix of Omnichrona [47]. Storage in tea and red wine produced the least roughness value, and this may be due to their acidic erosive effect [32]. The mean pH of tea and red wine were 4.9 [50] and 3.3 [51], respectively. It was reported that in low pH drinks, resin composites display high solubility, which causes surface dissolution [16] and possible re-precipitation, which may result in reduced surface roughness as shown in the AFM images (Fig. 1). Several reports are in agreement with the current findings, where low pH of the solution alone doesn’t indicate the aggressiveness of surface degradation of resin composites [8, 52]. Several other factors might contribute to the polymer’s degradation, including the crosslinking nature of the polymeric matrix, solubility parameter, and water uptake [52].

The intragroup comparison of storage media within the multi-shade group using the stylus profilometer did not detect any significant difference in the surface roughness among the various storage media. This may be due to its composition, which contains Bis-GMA and nanofillers. Bis-GMA provided lower susceptibility to acidic erosion and lower solubility compared to UDMA in the single-shade group [47, 48].

Using the AFM, the intragroup comparison of the storage media within the single-shade showed no significant difference in the nano-surface roughness. Within the multi-shade, the difference in surface roughness after the aging was less than 1 nm (maximum and minimum values were 1.3 nm and 0.5 nm, respectively). This minor variation in the surface roughness after artificial aging within each material may be attributed to the effect of the low pH drinks, which cause dissolution of the surface of resin composites and re-precipitation, resulting in nearly the same surface topography.

Thermocycling may increase water sorption, which may affect the mechanical properties of composites due to hydrolytic degradation and failure at the matrix-resin interface [17, 18]. In addition, thermocycling was reported to increase the surface degradation of resin composites due to the induction of superficial stresses and the formation of microcracks [53]. However, in the current study, no significant difference was detected in the surface roughness of specimens thermocycled for 10,000 cycles and non-thermocycled specimens. Similarly, other studies reported no significant difference in surface roughness following thermocycling compared to baseline [17, 53]. On the other hand, these findings were not in accordance with the study conducted by Minami et al. [17], where thermocycling for 50,000 cycles significantly increased the surface roughness of the investigated resin composites. These differences in results could be attributed to the difference in the thermocycling parameters employed in the different studies, where a higher number of cycles, simulating longer clinical durations, may increase the effect of thermocycling on the degradation of resin composites [17].

The surface roughness after acidic beverages depends on several factors, including the organic matrix structure and inorganic filler content and size, in addition to the characterization tool [32]. The chemical composition and pH of the acidic drink as well as the method of aging process performed also affect the results. This may explain the great variation in the literature studying the effect of acidic beverages on the surface roughness of resin composites. In the present research the surface roughness was assessed by two methods after two artificial aging protocols using three storage media for two resin composites with different organic composition and filler size. Despite these variables, it was observed in this study that all the reported surface roughness was clinically accepted. This may be due to the major effect of the submicron and nano-fillers.

Limitations of the current study include evaluating a limited number of materials. Using a variety of materials from different manufacturers with different compositions could be more comprehensive. In addition, although a Mylar strip was used to obtain a flat smooth surface, it was reported that it may produce a superficial layer reach in organic matrix, which could be more susceptible to staining and degradation [54]. However, in the current study this emphasized the importance of the matrix composition, where multi-shade composite containing Bis-GMA showed significantly lower surface nano-roughness compared to Bis-GMA free single-shade composite. Yet, future studies investigating the effect of artificial aging for longer durations following specimens’ finishing and polishing are needed to complement the results of the current study. Further studies are also required to investigate the long-term degradation effect of the nano-roughness, which may act as retentive areas for plaque, bacteria or food debris affecting the surface and longevity of dental restorations. Therefore, detecting a nano-roughness threshold/critical value could be beneficial.

## Conclusion

Within the limitation of the current study, the single shade (Omnichroma) and the multi-shade (Filtek Z350 XT) retained their surface finish with surface roughness values not exceeding the critical value of 0.2 μm after aging in artificial saliva, tea and red wine when measured by the surface profilometer and AFM.

## Data Availability

All data generated or analyzed during this study are included in this published article in the form of tables and figures. The raw data that support the findings of this study are available upon request from the corresponding author.
